# Detection of a New Recombinant Rabbit Hemorrhagic Disease Virus 2 in China and Development of Virus-like Particle-Based Vaccine

**DOI:** 10.3390/v17050710

**Published:** 2025-05-16

**Authors:** Bo Hu, Wenyu Dong, Yanhua Song, Zhiyu Fan, Patrizia Cavadini, Fang Wang

**Affiliations:** 1 Key Laboratory of Veterinary Biologicals Engineering and Technology, Institute of Veterinary Medicine, Jiangsu Academy of Agricultural Sciences, National Center for Engineering Research of Veterinary Bio-Products, Ministry of Agriculture, Nanjing 210014, China; hubolshg@163.com (B.H.); songyanhua8507@126.com (Y.S.); zy_fan1982@126.com (Z.F.); 2 College of Animal Science, Xizang Agriculture and Animal Husbandry College, Linzhi 860000, China; m18722279527@163.com; 3 GuoTai (Taizhou) Center of Technology Innovation for Veterinary Biologicals, Taizhou 225300, China; 4 WOAH Reference Laboratory for Rabbit Haemorrhagic Disease, Istituto Zooprofilattico Sperimentale della Lombardia e dell’Emilia Romagna, 25124 Brescia, Italy

**Keywords:** rabbit hemorrhagic disease virus, GI.2, recombination, VP60, China

## Abstract

Rabbit hemorrhagic disease virus (RHDV) is a very virulent virus of the genus Lagovirus causing severe and fatal hepatitis in the European rabbit (*Oryctolagus cuniculus*). RHDV has two distinct genotypes: GI.1 (RHDV) and GI.2 (RHDV2). The first RHDV2/GI.2 strain was identified as a recombinant virus between a non-pathogenic (GI.3P) and a pathogenic (GI.2) lagovirus, and the recombination is thought to have been a key mechanism in the emergence and evolution of RHDV2. Here, a new variant of RHDV2 was identified affecting domestic rabbits on Chinese farms, with a mortality rate of 70–80%. Phylogenetic analysis indicated that the nonstructural portion of this newly identified strain’s genome clustered with the GI.1a variants. In contrast, the capsid gene shared the highest nucleotide identity of 97.9% with the North American GI.2 strains, suggesting a possible introduction in China of North American strains and recombination with the GI.1a strains circulating in China. We have produced a recombinant vaccine using the first Chinese GI.2 strain, SC2020/0401, by cloning the vp60 gene into a baculovirus expression vector. Virus-like particles (VLPs) were then produced in Sf9 insect cells, and a challenge study was performed. Rabbits immunized with the VLP vaccine survived 7 d after being challenged with the new virus. The results indicate that commercial vaccines are urgently required in China to control the circulation of RHDV2 variants.

## 1. Introduction

Rabbit hemorrhagic disease virus (RHDV) is a single-stranded positive-sense RNA virus belonging to the family Caliciviridae, genus Lagovirus. Lagoviruses are classified based on the nucleotide capsid protein sequences (VP60) into genogroups (e.g., GI and GII), genotypes (e.g., GI.1, GI.2, GI.3, and GI.4), and variants (e.g., GI.1a, GI.1b, and GI.1c) [1]. Within this viral genus, pathogenic hepatotropic viruses (RHDV/GI.1 and RHDV2/GI.2) cause acute, fulminant viral hepatitis, whereas enterotropic viruses such as rabbit caliciviruses (GI.3/4) are completely benign. Rabbit hemorrhagic disease is highly contagious in the European rabbit (*Oryctolagus cuniculus*) with 100% morbidity and 80–95% lethality. It first emerged in 1984 in China [2] and then rapidly diffused worldwide in countries where the European rabbit is present.

RHDV2/GI.2 was first detected in France in 2010 [3] and spread all over Europe in a few years, and now it is present worldwide, including Australia [4,5], Africa [6,7], and North and Central America [8,9], where it has become endemic. Moreover, in China, RHDV2/GI.2 was reported in Sichuan province in April 2020 [10] and subsequently spread to other provinces, causing severe economic losses in the rabbit industry. The genome of the first RHDV2/GI.2 strain identified in China (GenBank accession number: MT586027) presented a high nucleotide sequence identity (98.3%) with the European RHDV2/GI.2 strain NL2016 (GenBank accession number: MN061492) [11]. Traceability research suggests that smuggling rabbit semen from Europe might be the cause of viral entry in China [12]. Previous studies have demonstrated that recombination plays a key role in the emergence and evolution of lagoviruses as has been demonstrated for RHDV2 [13,14,15,16]. Several recombination events have been reported for RHDV2/GI.2 between the nonstructural part of the genome and the structural part (vp60) originating from different genotypes (e.g., GI.1p-GI.2, GI.3p-GI.2, GI.4p-GI.2, and GII.1p-GI.2) [13,17,18,19]. In 2023, a novel recombinant strain was detected in China, with GI.1a serving as the major donor for the nonstructural part, whereas the GI.2 strain contributed to the structural part [20,21].

The predominant vaccines utilized against RHDV in China are the GI.1a vaccines [10], including inactivated tissue vaccines, and VP60-based subunit vaccines produced using insect cell expression systems. These vaccines have been effective in controlling outbreaks caused by the GI.1a strain of the virus, but fail to provide effective protection against the emerging GI.2 genotype [10]. This is due to substantial antigenic differences between the two viruses, allowing the GI.2 strains to evade immunity conferred by the current GI.1 vaccines [22]. Currently, the GI.2 genotype has become the dominant circulating strain in rabbit farms in China [23], emphasizing the urgent need for an effective vaccine specifically targeting this genotype. In addition, due to the lack of a cell culture system for RHDV propagation, most of the commercially available vaccines are based on inactivated infectious viruses from the liver suspensions of experimentally infected rabbits. Recombinant vaccines have been developed to address the ethical and animal welfare concerns associated with conventional liver-derived vaccine production. In this context, the development of VP60-based subunit vaccines is essential to improve toward ethically sustainable and efficacious disease control.

The RHDV capsid protein VP60 can self-assemble into virus-like particles (VLPs) and induce sufficient immunoprotection against RHDV infection [24,25]. The nucleotide diversity of VP60 between RHDV2 and RHDVa (GI.1a) is greater than 16% [26]. The two serotypes of RHDV have limited cross-protection, and the bivalent vaccine against both GI.1 and GI.2 RHDV is fully protective [27,28,29]. However, the protective capacity of the recombinant vaccine produced in China against RHDV2 with different viral introductions is not fully understood.

Therefore, this study aims to identify and characterize a new RHDV2 recombinant and determine the protective capacity provided by the VP60-VLP vaccine against it.

## 2. Materials and Methods

### 2.1. Sample Collection and Genotype Detection

In 2024, five liver samples were collected from two adjacent domestic rabbit farms in Henan province, China. The samples were stored at −70 °C. Total RNA was extracted from the liver samples using RNAiso plus (Takara, Dalian, China), followed by reverse transcription using the Reverse Transcriptase XL (AMV) kit (Takara) and Ex Taq kit (Takara). Here, two sets of specific primers targeting the VP60 gene were designed and used to identify the GI.1 and GI.2 genotypes of the RHDV. The GI.1 primers were RHDV-F (5′-TATTCTGGGAACAACTCCAC-3′) and RHDV-R (5′-AACAGTCCGGTTGGATTTTG-3′) that amplified a product of 347 bp. The GI.2 primers were RHDV2-F (5′-CCCTGGAAGCAGTTCGTCAAAC-3′) and RHDV2-R (5′-GATGTCAACAAGGTCTGACAG-3′) that amplified a product of 748 bp. Eight overlapping fragments of the complete genome sequence were amplified as previously described [13] and cloned into the pMD-19T vector (Takara). Sanger sequencing was performed, and complete genome sequences were obtained.

### 2.2. Phylogenetic Analysis

Phylogenetic analysis based on 135 lagoviruses sequences including all the available GI.1a/RHDVa sequences (62 strains) referred to the genes encoding for the nonstructural proteins (NSP: nucleotides 10–5304) and 131 referred to the genes encoding for structural proteins (SP: nucleotides 5305–7378) of the genome were performed using MEGA 7 (version 7.0.26) [30] with the maximum-likelihood approach based on the GTR+G+I model. The reliability of the nodes was assessed with a bootstrap resampling procedure consisting of 1000 replicates of the ML trees.

### 2.3. Recombination and Sequences Analysis

The RDP (version 5.5) [31] and SimPlot (version 3.5.1) [32] software were used to visualize recombination events based on the 265 full genome sequences. Web BLAST (https://blast.ncbi.nlm.nih.gov/Blast.cgi [accessed on 10 February 2025]) and Sequence Demarcation Tool (version 1.2) [33] were used for sequence analysis.

### 2.4. Challenge Study

Two copies of the vp60 gene of the SC2020/0401 strain (GenBank accession number: MT586027) were cloned into the pFastBac Dual vector (Invitrogen, Waltham, MA, USA) under the pH and p10 promoters. The recombinant bacmid was generated by transforming the recombinant plasmid into DH10Bac cells (Invitrogen). The recombinant baculovirus was produced using the Bac-to-Bac baculovirus expression system (Invitrogen). Sf9 cells were infected with the recombinant baculovirus at a multiplicity of infection (MOI) of 1 at 27 °C for 96 h. A hemagglutination test was used to confirm the expression of recombinant VP60 protein. Subsequently, VP60 was purified by salt precipitation with 20% ammonium sulfate, and the precipitate was recovered, resuspended, and dialyzed overnight as previously described [34]. The formation of VP60-based VLPs was observed by transmission electron microscopy (TEM). Ten RHDV2-seronegative 8-week-old New Zealand rabbits were randomly divided into two groups of five animals each. The vaccinated group was injected subcutaneously with the VP60-based VLPs (50 μg/mL without adjuvant, 1 mL per rabbit). The control group was injected with PBS (1 mL per rabbit). Fourteen days later, the rabbits in each group were injected subcutaneously with 2 × 10^4^ hemagglutination units (HAU) [35] of strain HN2024/0221-01. The animals were observed for 7 d, and dying rabbits were euthanized by air embolism for survival calculations.

## 3. Results

### 3.1. Genotype Detection and Phylogenetic Analysis

The total RNA extracted from the five liver samples was analyzed using RT-PCR as described in Section 2. Three of the five liver samples tested positive for RHDV2. Full-length genome sequences were obtained for three strains collected from Henan Province: HN2024/0221-01, HN2024/0221-03, and HN2024/0221-04 (GenBank accession numbers: PP539001, PP539002, and PP539003, respectively). The three strains showed nucleotide identities between 99.6% and 99.8% in their genome sequences, suggesting that they belonged to the same strain.

**Figure 1:**
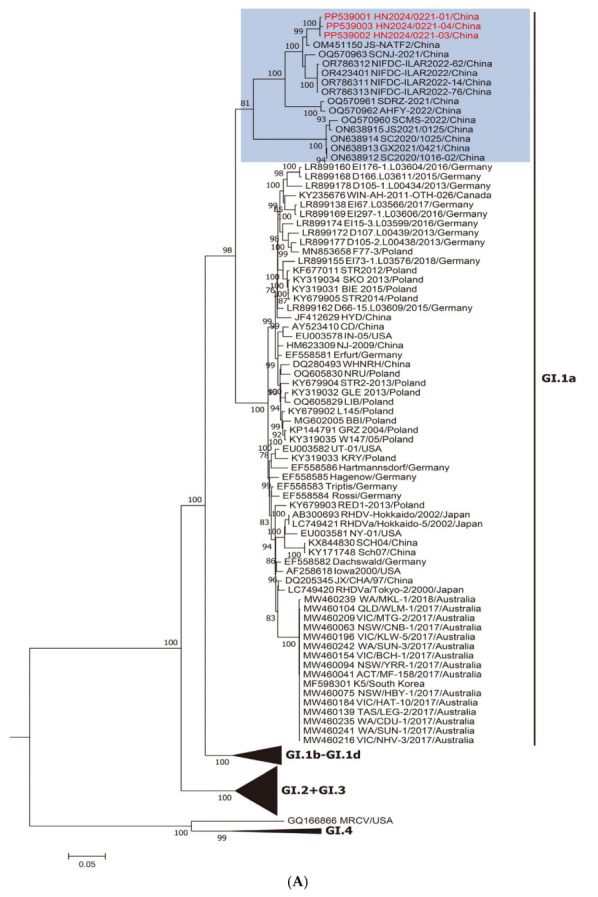

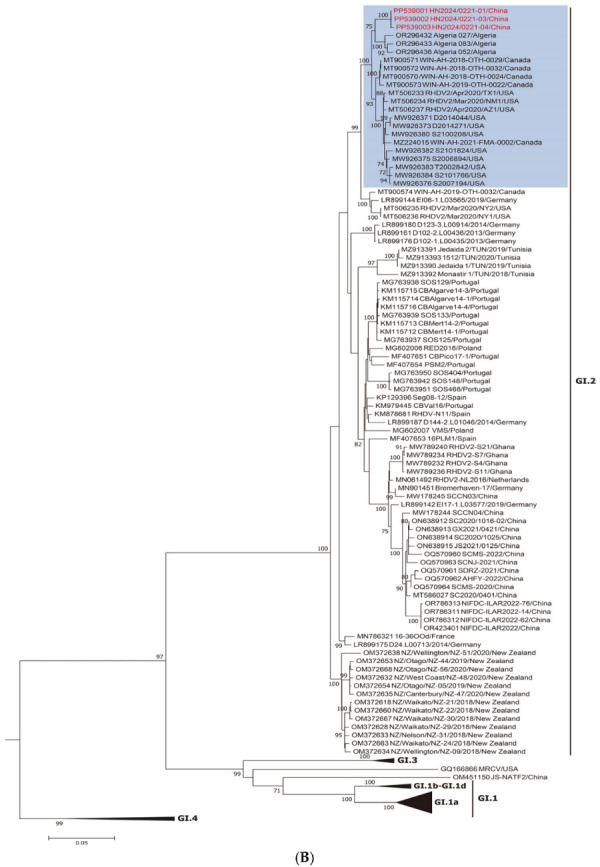
Maximum-likelihood (ML) phylogenetic trees for the genome regions defined by the recombination analysis. Bootstrap probability values above 70% with 1000 replicates are indicated at the nodes: (**A**) ML tree based on the nonstructural part of the genome (nucleotides 10–5304). The group including the new strains obtained in this study and the Chinese strain JS-NATF2 are shaded in blue. (**B**) ML tree based on the structural part of the genome (nucleotides 5305–7378). The group including the new strains obtained in this study and the USA GI.2 strain RHDV2/Apr2020/AZ1 are shaded in blue. GenBank accession numbers of the sequences are indicated in the taxon names. The European brown hare syndrome virus (EBHSV) strain GD (Z69620) was used as the outgroup to root the tree.

Two different ML trees were constructed: one based on NSP genes (nucleotide positions 10–5304; Figure 1A) and the other on SP genes (nucleotide positions 5305–7378; Figure 1B). As shown in Figure 1A, the new strains belong to the RHDVa (GI.1a) cluster for the nonstructural part of the genome. By contrast, as shown in Figure 1B, when the vp60 genes are considered, these strains belong to the RHDV2 (GI.2) cluster, suggesting a recombination event between GI.1a and GI.2 strains. This was a well-supported group (bootstrap value of 100; shaded blue).

### 3.2. Recombination and Sequences Analysis

**Figure 2:**
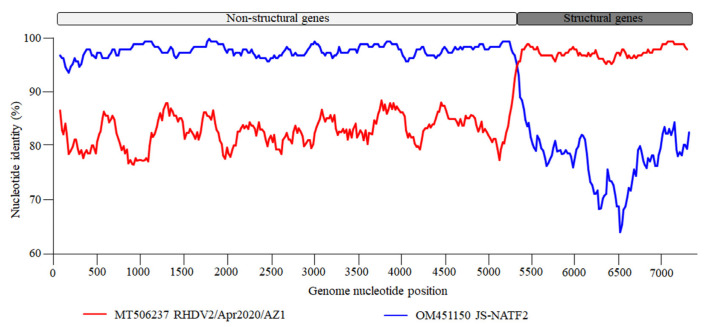
Genome recombination analysis of the HN2024/0221-01 strain. The SimPlot software (version 3.5.1) was used for analyzing the HN2024/0221-01 recombinant strain. The vertical axis represents the similarity to the two putative parental strains and the horizontal axis indicates the nucleotide positions. The blue plot indicates the closest major parent, JS-NATF2, donor of the NS portion of the genome, whereas the red plot indicates RHDV2/Apr2020/AZ1 strain, the closest minor parent, donor of the structural portion of the genome.

**Figure 3:**
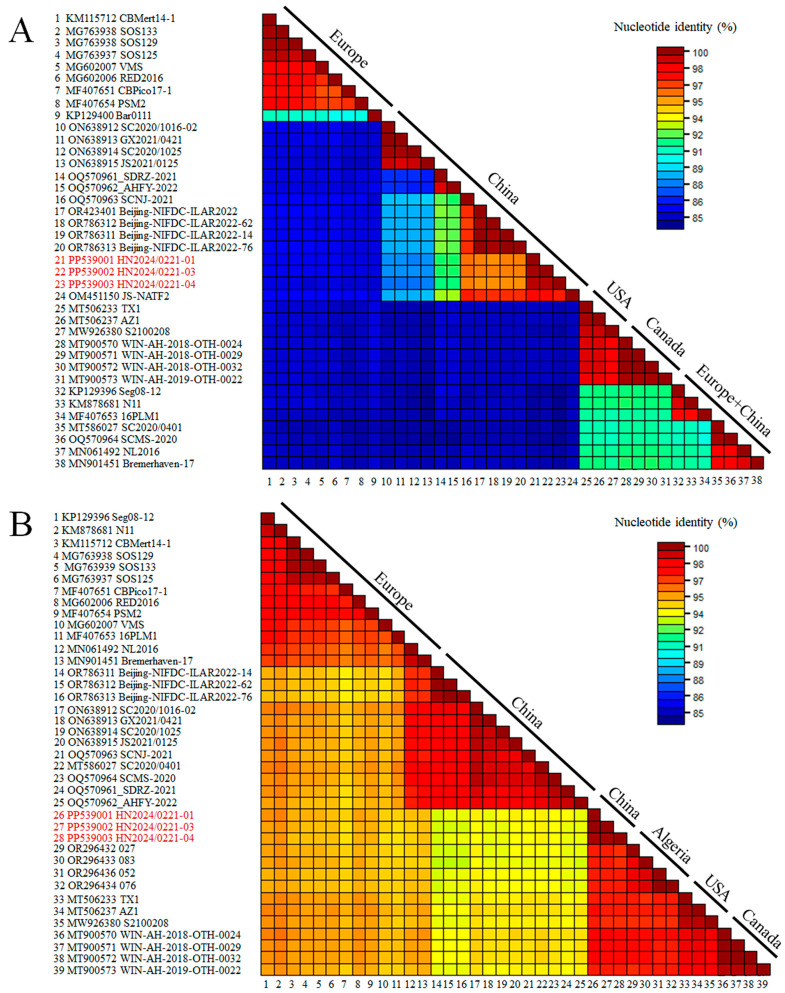
Nucleotide sequence analysis of the GI.2 strain compared to 39 sequences of lagoviruses present in GenBank: (**A**) The nonstructural part of the genome was considered (nucleotides 10–5304). (**B**) The structural part (nucleotides 5305–7378) was considered. GenBank accession numbers of the sequences are indicated close to the virus names.

The recombinant analysis confirmed that the new strain identified was a recombinant between an RHDVa (GI.1a) strain as a donor for the NS portion of the genome and an RHDV2 (GI.2) strain as a donor for the SP of the genome. The recombination breakpoint was detected with strong statistical evidence (*p* < 0.01) using the RDP, GENECONV, BootScan, MaxChi, Chimaera, SiScan, and 3Seq methods implemented in the RDP software (version 5.5) (Table 1), and was located at the boundary of the nonstructural/structural regions (positions 5170–5255; 99% confidence interval is indicated), as already shown for all RHDV2 [16]. The Chinese strain, JS-NATF2 (OM451150), was the most likely donor of the NSP of the genome, whereas the American strain, RHDV2/Apr2020/AZ1 (MT506237), was the most likely donor of the structural part of the genome. A recombination event between the Chinese and American strains was also confirmed by the SimPlot analysis (Figure 2). The BLAST analysis (https://blast.ncbi.nlm.nih.gov/Blast.cgi [accessed on 10 February 2025]) revealed that the five strains with the highest NSP nucleotide identity with the new isolate HN2024/0221-01 were JS-NATF2 (97.8%), SCNJ-2021 (96.2%), Bei-jing-NIFDC-ILAR2022-76 (95.9%), Beijing-NIFDC-ILAR2022-14 (95.9%), and Bei-jing-NIFDC-ILAR2022 (95.9%). All these strains were identified in China (Figure 3A). The five strains with the highest SP nucleotide sequence identity to HN2024/0221-01 were BC/Canada/WIN-AH-2018-OTH-0024 (97.9%), BC/Canada/WIN-AH-2018-OTH-0032 (97.8%), BC/Canada/WIN-AH-2018-OTH-0029 (97.8%), RHDV2/Apr2020/AZ1 (97.6%), and Algeria 076 (97.5%). These strains were collected from Canada, the USA, and Algeria (Figure 3B). However, the Algerian strains have been reported to have been introduced from North American countries [36].

### 3.3. Generation of RHDV2 VLPs and Challenge Study

**Figure 4:**
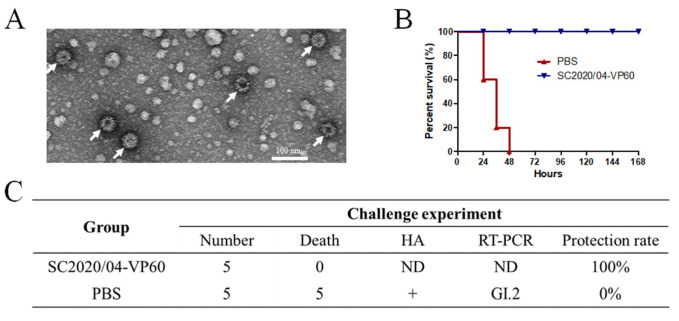
Protective efficacy of SC2020/0401 VP60-based VLPs against the HN2024/0221-01 strain: (**A**) Analysis of the SC2020/0401 VP60-based VLPs. The sample was negatively stained with 2% uranyl acetate and observed at ×40,000 magnification using a transmission electron microscope. The VLPs are shown by arrows. (**B**) Survival of the immunized and control rabbits infected with the HN2024/0221-01 strain. All the rabbits were clinically examined daily for 7 d. (**C**) Challenge results of the rabbits infected with the HN2024/0221-01 strain. HA and RT-PCR tests were used to identify the liver tissues from the dead rabbits.

Animal experiments were performed to determine the pathogenicity of the new strains and the protective efficacy of the VLP vaccine produced with recombinant VP60 from the first Chinese GI.2 strain SC2020/0401. The amino acid identity of the VP60 protein between HN2024/0221-01 and SC2020/0401 was found to be 97.2%. An amino acid sequence analysis demonstrated that there was some variability in the VP60 proteins. To increase VP60 protein expression, two copies of the vp60 gene from the SC2020/0401 strain under pH and p10 promoters in the pFastBac Dual vector were produced. VP60 was expressed in the Sf9 cells and purified. The TEM results showed that the VP60 protein formed VLPs, which were approximately 40 nm in diameter (Figure 4A). The protective efficacy of the vaccine based on SC2020/0401-VP60 VLPs against the new isolate, HN2024/0221-01, was evaluated 14 d after immunization. The results showed that isolate HN2024/0221-01 induced 100% mortality within 48 h in the group inoculated only with PBS, whereas the immunized group exhibited a 100% protection rate after 7 d (Figure 4B). HA and RT-PCR were used to detect the virus in the dead rabbits. The results showed that the liver samples from the PBS group tested positive for hemagglutination and GI.2 RNA (Figure 4C). The rabbits inoculated with VLPs showed complete immune protection against the virus challenge with HN2024/0221-01 infection.

## 4. Discussion

The RHDV2 virus was first identified in China in 2020 [10]. Sequence and evolutionary analyses revealed that the Chinese strains were closely related and highly similar to the European strains [11]. New recombinant RHDV2 strains were detected in China within one year of the original RHDV2 arrival [20], highlighting the remarkable recombination ability of RHDV2. The structural genes of new recombinants clustered with the first Chinese RHDV2 SC2020/0401 strain and the European strains, whereas the nonstructural portion of the genome was in the same branch as the GI.1a strains [20,21]. Virus traceability studies have also suggested that the introduction of the original RHDV2 into China may be related to rabbit semen imported from Europe [12].

The first known case of RHDV2 infection in Canada occurred in 2016, followed by several outbreaks in Canada and the USA [9]. Additionally, Algerian isolates collected between 2020 and 2021 were found to be closely related to North American RHDV2 isolates [36]. In this study, the structural genes of the three RHDV2 strains identified in China in 2024 clustered with those of the Canadian and American strains, suggesting a possible introduction of North American strains. Although the route of viral entry remains unclear, the introduction of North American RHDV2 may be linked to trade in agricultural products. China is one of the top countries in global rabbit breeding, and trade between these countries constitutes the majority of global rabbit product commerce [37]. These results suggested the existence of multiple routes of entry of RHDV2 into China.

Recombination is recognized as an important factor driving lagovirus evolution [38] and several recombinants have been described for RHDV2 [13,17,18,19]. In this study, we have described a new recombinant where the nonstructural portion of the genome clustered with the Chinese GI.1a strains (Figure 1A; shaded blue), indicating that these strains contributed to the nonstructural part of the new virus. More specifically, the likely major donor strain should be JS-NATF2 (Figure 1A,B), which has been previously identified in wild rabbits in China by meta-transcriptomic analysis [39], and has been reported to contribute nonstructural genes to other Chinese recombinant strains [20]. Based on the recombination analysis performed in this study, it is proposed that the formation of the new strains involved a recombination event between the North American GI.2 strain and the Chinese GI.1a strain. This is the first study to report the detection of a novel recombinant RHDV2 strain in China with the North American RHDV2 strain contributing to the structural genes. Although it is not clear if the recombination event occurred in the Americas or China, the emergence of this virus suggests the introduction in China of North American strains.

Several expression systems have been used for RHDV2 VP60 capsid protein expression, including recombinant baculoviruses, mammalian cells, and yeasts [27,40,41]. Some of them have already been commercialized, and their efficacy against different virulent strains of RHDV2 has been demonstrated [42,43]. The baculovirus-expressed structural protein VP60 of RHDV2 can form VLPs with sizes ranging from 30 to 40 nm [44] and protect against lethal doses of the virus [28]. In this study, two copies of the vp60 gene under two promoters (pH and p10) in the pFastBac Dual vector were produced to increase VP60 protein expression. This construction method was used for the expression of PPRV proteins for VLP production in a previous study [45]. The VP60 protein of the SC2020/0401 strain was expressed in the Sf9 cells and formed VLPs of approximately 40 nm in size. To evaluate the protective efficacy of VP60-based VLPs against the new recombinant virus HN2024/0221-01 with structural genes from North American strains, New Zealand rabbits were challenged with this virus 14 d after immunization with the VLP vaccine.

In our challenge tests, all the control rabbits died within 48 h, indicating the high virulence of this new strain. Encouragingly, the VLP vaccine used in this study provided complete protection against the new recombinant strain collected in 2024, suggesting that the VLP vaccine could protect against infection with heterologous strains of different origins and certain variability in the vp60. Given the potential circulation of several RHDV2 strains in China, commercial vaccines are urgently required to control RHDV2 variants.

## Figures and Tables

**Table 1:** Recombination analysis of Chinese RHDV2 strains using RDP5 software.

Strains	Most Likely Donor Strain		Methods and Average *p*-Values						
	NSP ^1^	SP ^2^	RDP	GENECONV	BootScan	MaxChi	Chimaera	SiScan	3Seq
HN2024/0221-01 (PP539001)	JS-NATF2 (OM451150)	AZ1 (MT506237)	2.145 × 10^−46^	3.002 × 10^−109^	1.671 × 10^−112^	2.545 × 10^−37^	2.381 × 10^−39^	4.786 × 10^−43^	1.912 × 10^−174^
HN2024/0221-03 (PP539002)									
HN2024/0221-04 (PP539003)									

^1^ nonstructural proteins. ^2^ structural proteins.

## Data Availability

All the viral genome sequences in this study were submitted to the National Center for Biotechnology Information (NCBI) under GenBank accession numbers PP539001-PP539003.

## Abbreviations

The following abbreviations are used in this manuscript:

RHD	rabbit hemorrhagic disease
RHDV	rabbit hemorrhagic disease virus
VLPs	virus-like particles
ML	maximum likelihood
TEM	transmission electron microscope
HAU	hemagglutination units
EBHSV	European brown hare syndrome virus
